# Physician perspectives of Helicobacter pylori diagnostic and treatment practices in Canada: results of a Canadian survey

**DOI:** 10.1186/s12876-024-03293-w

**Published:** 2024-06-17

**Authors:** Kianna Cadogan, Seth R. Shaffer, Alexander Singer, Aleisha Reimer, Natalie Knox, Jillian Rumore, Sara Christianson, David C. Alexander, Jessica D. Forbes, Charles N. Bernstein

**Affiliations:** 1 Department of Internal Medicine, Rady Faculty of Health Sciences, Max Rady College of Medicine, University of Manitoba, Winnipeg, MB Canada; 2 Section of Gastroenterology, Department of Internal Medicine, Rady Faculty of Health Sciences, Max Rady College of Medicine, University of Manitoba, 804-715 McDermot Avenue, Winnipeg, MB R3E3P4 Canada; 3 Department of Family Medicine, Rady Faculty of Health Sciences, Max Rady College of Medicine, University of Manitoba, Winnipeg, MB Canada; 4 National Microbiology Laboratory Branch, Public Health Agency of Canada, Winnipeg, MB Canada; 5 Cadham Provincial Laboratory, Diagnostic Services, Shared Health, Winnipeg, MB Canada; 6 Department of Medical Microbiology & Infectious Diseases, Rady Faculty of Health Sciences, University of Manitoba, Winnipeg, MB Canada; 7 Eastern Ontario Regional Laboratory Association, Ottawa, ON Canada; 8 Department of Pathology and Laboratory Medicine, University of Ottawa, Ottawa, ON Canada

**Keywords:** Helicobacter pylori, Peptic ulcer disease, Antibiotic resistance

## Abstract

**Background:**

*Helicobacter pylori* infection is prevalent worldwide and can lead to peptic ulcer disease (PUD) and gastric cancer. Effective diagnosis and treatment of *H. pylori* infection by gastroenterologists and family physicians is crucial. However, there are differing views on optimal diagnosis and treatment. The objective of this study is to understand the impressions of Canadian physicians regarding *H. pylori* diagnosis and treatment and whether impressions differ between gastroenterologists and family physicians. A second objective is to understand physician perspectives on rising antibiotic resistance and how that guides empiric management.

**Methods:**

A survey facilitated via REDCap was administered to Canadian gastroenterologists and family physicians. A total of 105 participants completed the survey, including 43 gastroenterologists and 62 family physicians. Gastroenterologists were recruited from across the country and family physicians were recruited from Manitoba.

**Results:**

For diagnosis of *H. pylori*, 67% of gastroenterologists reported endoscopic biopsies for histology assessment as most common and 73% of family physicians reported serology as their main diagnostic test. While nearly all gastroenterologists believed antibiotic resistance to be a problem, nearly one quarter of family physicians did not believe it was a problem.

**Conclusions:**

There is variability in practices among both gastroenterologists and family physicians regarding diagnosis of *H. pylori* infection. There was consensus that local antibiotic resistance patterns should guide management. If known, the degree and patterns of antibiotic resistance could bring a more uniform consensus to *H. pylori* management. Greater education of physicians, especially family physicians regarding management of H pylori is needed.

## Background

*Helicobacter pylori* infection is prevalent worldwide and if left untreated can lead to peptic ulcer disease (PUD) and gastric cancer [1, 2]. It is responsible for over 80% of cases of gastric cancer [3, 4]. Diagnosis and treatment of *H. pylori* infection is thus crucial for gastroenterologists and family physicians. However, there are differing views on optimal diagnosis and treatment. For instance, a Canadian study in 2007 found differences in inpatient and outpatient treatments of *H. pylori* infection in London, Ontario with inpatients generally being undertreated compared to outpatients [5]. There are also increasing concerns of antibiotic resistance to common antibiotic regimens used to eradicate *H. pylori* which affects optimal empiric management [6–9]. Current guidelines recommend using local antibiotic resistance patterns to guide empiric treatment of *H. pylori* infection [3]. While these guidelines exist, access to antibiotic resistance patterns are limited in North America [1, 10]. The lack of antimicrobial resistance patterns for *H. pylori* is primarily due to (i) the widespread use of culture-independent diagnostic tests that preclude phenotypic antimicrobial susceptibility testing and (ii) limited access to both phenotypic and molecular methods for *H. pylori* susceptibility testing.

Previous studies have outlined differing perspectives on *H. pylori* treatment. A 2003 study surveyed primary care physicians in countries around the world and found that there were contrasting approaches to diagnosing and treating *H. pylori* infection depending on the country where the providers practiced. This study also showed that despite quadruple therapy being the main recommendation for *H. pylori* treatment, most survey respondents had provided treatment with triple therapy regimens [11]. A study in 1998 surveyed Canadian physicians and determined that there were many different strategies used to manage new-onset dyspepsia in the primary care setting [12]. It is unclear from the literature whether current guidelines for *H. pylori* diagnosis and management are being applied in Canada. It is also unclear whether perspectives and management differs between gastroenterologists and family physicians, both of whom regularly diagnose and manage *H. pylori* infections. The objective of our study is to better understand the impressions of Canadian physicians regarding *H. pylori* diagnosis and treatment and whether the impressions are different between gastroenterologists and family physicians. A second objective is to understand physician perspectives on rising antibiotic resistance and how that guides empiric management.

## Methods

A survey facilitated via REDCap [13, 14] was administered to gastroenterologists across Canada and family physicians in Manitoba. Participation in the survey was voluntary and informed consent was obtained from all participants. There were two sections of the survey: basic demographics/background information and *H. pylori* diagnostic/treatment approach. Results from the survey were collected and no personal or identifying information was collected from participants. A total of 105 participants completed the survey. Gastroenterologists were accessed by contacting the Division Heads of the Divisions of Gastroenterology at University of British Columbia, University of Alberta, University of Calgary, University of Saskatchewan, University of Manitoba, Western University, University of Toronto, University of Ottawa and McGill University to distribute to their division members. Family physicians were contacted through the University of Manitoba Department of Family Medicine. Research Ethics Board approval was obtained from the University of Manitoba Research Ethics Board (HS25538).

## Results

**Fig. 1:**
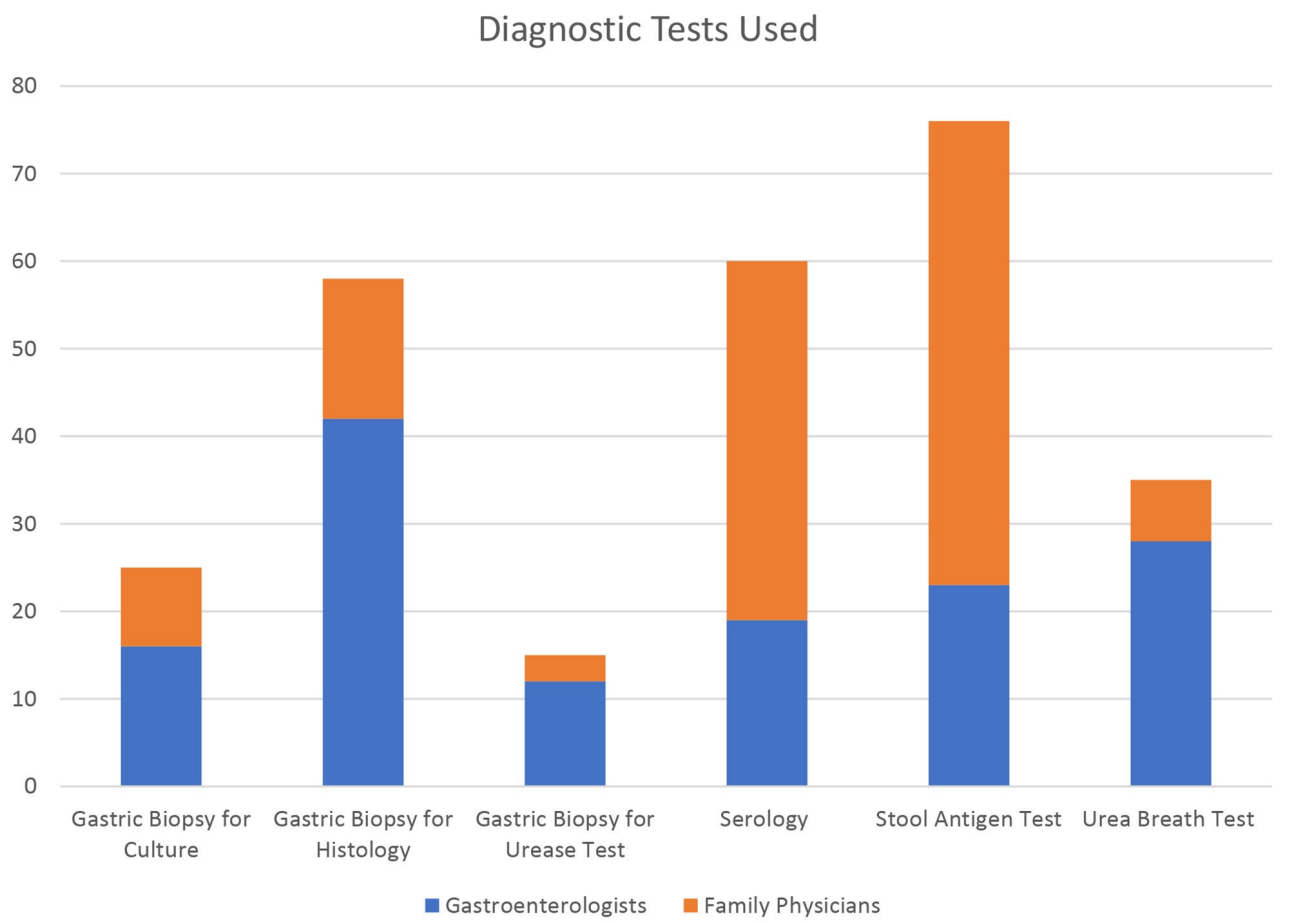
Diagnostic Tests Used

A total of 43 gastroenterologists and 62 family physicians completed the survey. The gastroenterologists were distributed across Canada while the family physicians were limited to Manitoba. 60% of gastroenterologists practiced exclusively at tertiary hospitals while 60% of family physicians worked exclusively in community practice (Table 1). Most respondents thought that less than half of peptic ulcer cases within the last year were associated with *H. pylori*. Among gastroenterologists, 25% do not routinely biopsy for *H. pylori* during upper endoscopy for any reason. Just over one third of family physician respondents only test for *H. pylori* eradication after treatment less than 25% of the time. Nearly three quarters of respondents use stool antigen testing to diagnose *H. pylori*, while over half use serology (Fig. 1). In terms of the most used tests for diagnosis of *H. pylori*, 67% of gastroenterologists reported endoscopic biopsies for histology assessment as most common and 73% of family physicians reported serology as their main diagnostic test. Over one quarter of physicians believe that the optimal method for diagnosing *H. pylori* is gastric biopsy for culture, followed by stool antigen test (19.1%), and urea breath testing (18.1%) (Table 2).

**Table 1:** Physician Demographics

	Total (*n* = 105)	Gastroenterologists (*n* = 43)	Family Physicians (*n* = 62)
**Gender**			
Female	50 (47.6%)	9 (20.9%)	41 (66.1%)
Male	51 (48.6%)	33 (76.7%)	18 (29.0%)
Unspecified	4 (3.8%)	1 (2.3%)	3 (4.8%)
**Ethnicity**			
Caucasian	70 (66.7%)	27 (62.8%)	43 (69.4%)
Black	4 (3.8%)	N/A	4 (6.5%)
Middle Eastern	5 (4.8%)	3 (7.0%)	2 (3.2%)
Latin American	3 (2.9%)	2 (4.7%)	1 (1.6%)
Asian	13 (12.4%)	9 (20.9%)	4 (6.5%)
Other	3 (2.9%)	1 (2.3%)	2 (3.2%)
Prefer not to Answer	7 (6.7%)	1 (2.3%)	6 (9.7%)
**Age**			
30–39	28 (26.7%)	10 (23.3%)	18 (29.0%)
40–49	36 (34.3%)	14 (32.6%)	22 (35.5%)
50–59	26 (24.8%)	13 (30.2%)	13 (30.0%)
60+	15 (14.3%)	6 (14.0%)	9 (14.5%)
**Year of MD Completion**			
< 5 years	5 (4.8%)	N/A	5 (8.1%)
5–9 years	20 (19.1%)	6 (14.0%)	14 (22.6%)
10–29 years	56 (53.3%)	27 (62.8%)	29 (46.8%)
30 + years	24 (22.9%)	10 (23.3%)	14 (22.6%)
**Location of Practice**			
Alberta	3 (2.9%)	3 (7.0%)	N/A
British Columbia	14 (13.3%)	14 (32.6%)	N/A
Manitoba	77 (73.3%)	15 (34.9%)	62 (100%)
Ontario	8 (7.6%)	8 (18.6%)	N/A
Quebec	2 (1.9%)	2 (4.7%)	N/A
Saskatchewan	1 (1.0%)	1 (2.3%)	N/A
**Primary Practice Setting**			
Community Practice	37 (35.2%)	1 (2.3%)	36 (58.06%)
Community Practice and Community Hospital	25 (23.8%)	4 (9.3%)	21 (33.87%)
Community Practice and Tertiary Hospital	9 (8.6%)	7 (16.3%)	2 (3.23%)
Community Practice and Community and Tertiary Hospital	5 (4.8%)	4 (9.3%)	1 (1.61%)
Emergency	1 (1.0%)	N/A	1 (1.61%)
Indigenous Rural Community and Inpatient Hospital	1 (1.0%)	N/A	1 (1.61%)
Tertiary Hospital	26 (24.8%)	26 (60.5%)	0 (0%)
Tertiary and Community Hospital	1 (1.0%)	1 (2.3%)	0 (0%)

**Table 2:** *H. pylori* Diagnostic Testing and Management

	Total (*n* = 105)	Gastroenterologists (*n* = 43)	Family Physicians (*n* = 62)
**Percent of Peptic Ulcer Cases Associated with ** ***H. pylori *** **Positivity in Past Year**			
< 25%	47 (44.8%)	14 (32.6%)	33 (53.2%)
25–49%	35 (33.3%)	17 (39.5%)	18 (29.0%)
50–74%	15 (14.3%)	9 (20.9%)	6 (9.7%)
75–99%	7 (6.7%)	3 (7.0%)	4 (6.5%)
100%	1 (1.0%)	N/A	1 (1.6%)
**Biopsy for H Pylori During Upper Endoscopy**			
< 25%	6 (5.7%)	6 (14%)	N/A
25–49%	5 (4.8%)	5 (11.6%)	N/A
50–74%	5 (4.8%)	5 (11.6%)	N/A
75–99%	12 (11.4%)	12 (27.9%)	N/A
100%	15 (14.3%)	15 (34.9%)	N/A
Not part of their practice	62 (59.1%)	N/A	62 (100%)
**% Follow-up Testing for Eradication**			
< 25%	23 (21.9%)	1 (2.3%)	22 (35.5%)
25–49%	13 (12.4%)	3 (7.0%)	10 (16.1%)
50–74%	8 (7.6%)	6 (14.0%)	2 (3.2%)
75–99%	20 (19.1%)	14 (32.6%)	6 (9.7%)
100%	26 (24.8%)	19 (44.2%)	7 (11.3%)
Only if ulcer present	15 (14.3%)	N/A	15 (24.2%)
**Percent Testing for ** ***H. pylori *** **in Patients with Upper GI Complaints**			
< 25%	18 (17.1%)	6 (14.0%)	12 (19.4%)
25–49%	21 (20.0%)	6 (14.0%)	15 (24.2%)
50–74%	24 (22.9%)	10 (23.3%)	14 (22.6%)
75–99%	34 (32.4%)	16 (37.2%)	18 (29.0%)
100%	8 (7.6%)	5 (11.6%)	3 (4.8%)
**Diagnostic Tests Used (Select All That Apply)**			
Gastric Biopsy for Culture	25	16	9
Gastric Biopsy for Histology	58	42	16
Gastric Biopsy for Urease Test	15	12	3
Serology	60	19	41
Stool Antigen Test	76	23	53
Urea Breath Test	35	28	7
**Diagnostic Tests Used Most Frequently**			
Gastric Biopsy for Culture	4 (3.8%)	3 (7.0%)	1 (1.6%)
Gastric Biopsy for Histology	31 (29.5%)	29 (67.4%)	2 (3.2%)
Gastric Biopsy for Urease Test	5 (4.8%)	3 (7.0%)	2 (3.2%)
Serology	46 (43.8%)	1 (2.3%)	45 (72.6%)
Stool Antigen Test	7 (6.7%)	5 (11.6%)	2 (3.2%)
Urea Breath Test	12 (11.4%)	1 (2.3%)	11 (17.7%)
**Optimal Diagnostic Test for ** ***H. pylori***			
Gastric Biopsy for Culture	28 (26.7%)	5 (11.6%)	23 (37.1%)
Gastric Biopsy for Histology	29 (27.6%)	18 (41.9%)	11 (17.7%)
Gastric Biopsy for Urease Test	7 (6.7%)	3 (7.0%)	4 (6.5%)
Serology	2 (1.9%)	N/A	2 (3.2%)
Stool Antigen Test	20 (19.1%)	7 (16.3%)	13 (21.0%)
Urea Breath Test	19 (18.1%)	10 (23.3%)	9 (14.5%)
***H. pylori *** **Treatment**			
Treat themselves	100 (95.2%)	40 (93.0%)	60 (96.8%)
Defer treatment to other physician	5 (4.8%)	3 (7.0%)	2 (3.2%)
**Preferred Duration of Therapy**			
7 days	6 (5.7%)	N/A	6 (9.7%)
10 days	9 (8.6%)	1 (2.3%)	8 (12.9%)
14 days	90 (85.7%)	42 (97.7%)	48 (77.4%)
**PPI Included In the Regimen**			
Yes	105 (100%)	43 (100%)	62 (100%)
No	0 (0%)	N/A	N/A

While nearly all gastroenterologists believed antibiotic resistance to be a problem, nearly one quarter of family physicians did not believe it to be a problem. Approximately half of family physicians thought there was resistance to amoxicillin in at least 25% of cases, while over half of gastroenterologists thought there was resistance to clarithromycin in at least 25% of cases (Table 3). Only one third of respondents would use antibiotic resistance profiles to guide treatment selection 100% of the time, with similar responses from both gastroenterologists and family physicians (Table 4).

**Table 3:** Physician Impression of *H. pylori* Antibiotic Resistance

	Total (*n* = 105)	Gastroenterologists (*n* = 43)	Family Physicians (*n* = 62)
**Is Antibiotic Resistance a Problem?**			
Yes	90 (85.7%)	42 (97.7%)	48 (77.4%)
No	15 (14.3%)	1 (2.3%)	14 (22.6%)
**Degree of Resistance: Amoxicillin**	*n* = 90	*n* = 42	*n* = 48
< 10%	18 (20.0%)	15 (35.7%)	3 (6.3%)
10–24%	39 (43.3%)	17 (40.5%)	22 (45.8%)
25–50%	29 (32.2%)	9 (21.4%)	20 (41.7%)
50+%	4 (4.4%)	1 (2.4%)	3 (6.3%)
**Degree of Resistance: Clarithromycin**	*n* = 90		
< 10%	10 (11.1%)	4 (9.5%)	6 (12.5%)
10–24%	40 (44.4%)	14 (33.3%)	26 (54.2%)
25–50%	24 (26.7%)	15 (35.7%)	9 (18.8%)
50+%	16 (17.8%)	9 (21.4%)	7 (14.6%)
**Degree of Resistance: Metronidazole**	*n* = 90		
< 10%	35 (38.9%)	13 (31.0%)	22 (45.8%)
10–24%	29 (32.2%)	14 (33.3%)	15 (31.3%)
25–50%	20 (22.2%)	12 (28.6%)	8 (16.7%)
50+%	6 (6.7%)	3 (7.1%)	3 (6.3%)

**Table 4:** Physician Impression of Further Antibiotic Susceptibility Testing Options

	Total (*n* = 105)	Gastroenterologists (*n* = 43)	Family Physicians (*n* = 62)
**How Often Would Antibiotic Susceptibilities on Gastric Biopsy Be Used?**			
< 25%	13 (12.4%)	10 (23.3%)	3 (4.8%)
25–49%	11 (10.5%)	5 (11.6%)	6 (9.7%)
50–74%	15 (14.3%)	7 (16.3%)	8 (12.9%)
75–99%	14 (13.3%)	8 (18.6%)	6 (9.7%)
100%	34 (32.4%)	13 (30.2%)	21 (33.9%)
Do not treat based on biopsy	18 (17.1%)	N/A	18 (29.0%)
**How Often Would Antibiotic Susceptibilities on Stool Testing Be Used?**			
< 25%	14 (13.3%)	10 (23.3%)	4 (6.5%)
25–49%	8 (7.6%)	6 (14.0%)	2 (3.2%)
50–74%	13 (12.4%)	5 (11.6%)	8 (12.9%)
75–99%	14 (13.3%)	6 (14.0%)	8 (12.9%)
100%	36 (34.3%)	11 (25.6%)	25 (40.3%)
Do not treat based on stool testing	20 (19.1%)	5 (11.6%)	15 (24.2%)
**Would Local Resistance Patterns Influence Management?**			
Yes	105 (100%)	43 (100%)	62 (100%)
No	0 (0%)	N/A	N/A

## Discussion

Our study found that there are substantial differences in approach to *H. pylori* management between gastroenterologists and family physicians. Some of the differences are driven by the ability of gastroenterologists to perform upper endoscopy as well as stool antigen testing being restricted to gastroenterologists (in Manitoba), while many family physicians rely heavily on serology despite its poor specificity. Amongst the more surprising findings were that most respondents thought that less than half of peptic ulcer cases within the last year were associated with *H. pylori* (the true proportion is greater than 85%), and that 65% of gastroenterologists do not always biopsy for *H. pylori* when they have the opportunity during upper endoscopy. Nearly all gastroenterologists believed antibiotic resistance to be a problem, yet nearly one quarter of family physicians did not believe it was a problem. It was also surprising that if available, only one third of respondents would use antibiotic susceptibility testing on gastric biopsy or stool testing to guide treatment selection 100% of the time. Hence, there is no consensus between gastroenterologists and family physicians on the optimal way to diagnose and manage *H. pylori* or to what extent there is a problem of antibiotic resistance in *H. pylori* management. Nonetheless, if local resistance patterns were available there was unanimity that local antibiotic resistance patterns would guide treatment.

The most recent guidelines on *H. pylori* management recommend testing for *H. pylori* in all patients with PUD, previous history of PUD, low-grade gastric mucosa-associated lymphoid tissue (MALT) lymphoma, or endoscopic resection of early gastric cancer. Additionally, they recommend testing for uninvestigated dyspepsia. The recommendations state that patients with typical symptoms of gastroesophageal reflux disease (GERD) with no history of PUD do not need to undergo *H. pylori* testing [1, 10, 16]. The results of our survey showed that participants did not always choose to test for *H. pylori* in their patients with upper gastrointestinal complaints with 37% of participants only testing less than half of the time (Table 2). The guidelines also recommend follow up testing for eradication four weeks after finishing treatment with either a urea breath test, stool antigen test, or biopsy-based testing [1, 10, 16]. Only one quarter of participants always performed follow-up testing for eradication (Table 2). Current guidelines do not make a recommendation about the optimal diagnostic test for *H. pylori* infection. Additionally, current guidelines recommend using patients’ previous antibiotic exposure as a factor in choosing an antibiotic regimen as well as considering local resistance patterns. Subsequently, there are differing recommended first line regimens based on antibiotic resistance patterns. Yet, in Canada availability of information on local antibiotic resistance to *H. pylori* is not routine, and if present, are rarely made easily available to treating physicians [1, 10, 16]. Antimicrobial resistance patterns can be determined utilizing culture methods or molecular testing; however, this is often not available in clinical settings [1]. Antimicrobial susceptibility testing of cultured isolates is not regularly performed due to challenges associated with the fastidious nature of *H. pylori* whereas interpretive criteria for molecular methods are generally limited to only clarithromycin resistance [1]. Future directions of research should focus on developing a culture-independent genomics-based approach to predict local antimicrobial resistance patterns and guide empiric treatment of *H. pylori* infection as well as providing antimicrobial susceptibility reference testing to personalize treatment when required.

A main limitation of the study is the modest sample size (*n* = 105). While the family physician respondents were practicing exclusively in Manitoba, the gastroenterology participants were drawn from across the country. Considering that the responses are quite varied it is more likely that the survey responses reflect the lack of consensus on the management of *H. pylori* in Canada.

Major discrepancies between gastroenterologists and family physicians exist despite guidelines and consensus reports that provide recommendations on H pylori management [15]. In some areas of management, many responses by gastroenterologist were not in keeping with the recent consensus conference report [15]. This further emphasizes the need for better education of both Canadian gastroenterologists and family physicians on the approach to *H pylori* management. In particular, the recent consensus conference on management of H pylori was published in a leading gastroenterology journal, one which may not be widely accessed by family physicians. Hence, an update on H pylori management needs to be disseminated in publications and through other communication vehicles more widely accessed by primary care providers.

## Conclusions

Infection with *H. pylori* is associated with PUD and gastric cancer [6]. There is variability in practices among both gastroenterologists and family physicians regarding who to test for *H. pylori* infection and which testing modality to use. There are also varying perceptions about whether follow-up testing for eradication is necessary. There was uniform consensus that local antibiotic resistance patterns should guide management. While antibiotic resistance patterns should guide empiric therapy, access to local resistance patterns is limited in Canada [1, 10, 16]. Greater consensus across Canadian physicians is needed regarding the management of *H. pylori* infection. If known, the degree and patterns of antibiotic resistance, supported by national guidelines for *H. pylori* diagnosis and treatment, could bring a more uniform consensus to *H. pylori* management in Canada and improve the awareness of both *H. pylori*’s significance in PUD and growing impact of antimicrobial resistance. Recent consensus guidelines and reports have been published on management of H pylori, but many practicing physicians are not adhering to these guidelines, underscoring a need for greater promotion of these guidelines more widely. This is especially true for family physicians, such that reporting of the most up to date approaches to H pylori management needs to be more widely accessible to them.

## Data Availability

Data are provided within the manuscript.

## Abbreviations

PUD: Peptic ulcer disease
MALT: Mucosa-associated lymphoid tissue
GERD: Gastroesophageal reflux disease
